# Diagnosing cervical lymph node metastasis in oral squamous cell carcinoma based on third-generation dual-source, dual-energy computed tomography

**DOI:** 10.1007/s00330-022-09033-6

**Published:** 2022-09-07

**Authors:** Yong-Heng Luo, Xi-Long Mei, Qin-Ru Liu, Bo Jiang, Sheng Zhang, Ke Zhang, Xia Wu, Yong-Mei Luo, Ya-Jun Li

**Affiliations:** 1 Department of Radiology, The Second Xiangya Hospital of Central South University, 139 Renmin Middle Road, Changsha, 410011 Hunan Province China; 2 Department of Stomatology, The Second Xiangya Hospital of Central South University, Changsha, 410011 Hunan Province China; 3 Department of Pathology, The Second Xiangya Hospital of Central South University, Changsha, 410011 Hunan Province China; 4 Department of Safety & Environmental Protection, Shenzhen Zhongjin Lingnan Nonfemet Company Ltd., Shenzhen, 518040 Guangdong China

**Keywords:** CT, Oral squamous cell carcinoma, Lymph node metastasis, Microvascular density

## Abstract

**Objectives:**

To investigate the potential of dual-energy computed tomography (DECT) parameters in identifying metastatic cervical lymph nodes in oral squamous cell carcinoma (OSCC) patients and to explore the relationships between DECT and pathological features.

**Methods:**

Clinical and DECT data were collected from patients who underwent radical resection of OSCC and cervical lymph node dissection between November 2019 and June 2021. Microvascular density was assessed using the Weidner counting method. The electron density (ED) and effective atomic number (*Z*_eff_) in non - contrast phase and iodine concentration (IC), normalized IC, slope of the energy spectrum curve (*λ*_HU_), and dual-energy index (DEI) in parenchymal phase were compared between metastatic and non - metastatic lymph nodes. Student’s *t*-test, Pearson’s rank correlation, and receiver operating characteristic curves were performed.

**Results:**

The inclusion criteria were met in 399 lymph nodes from 103 patients. Metastatic nodes (*n* = 158) displayed significantly decreased ED, IC, normalized IC, *λ*_HU_, and DEI values compared with non-metastatic nodes (*n* = 241) (all *p* < 0.01). Strong correlations were found between IC (*r* = 0.776), normalized IC (*r* = 0.779), *λ*_HU_ (*r* = 0.738), DEI (*r* = 0.734), and microvascular density. Area under the curve (AUC) for normalized IC performed the highest (0.875) in diagnosing metastatic nodes. When combined with the width of nodes, AUC increased to 0.918.

**Conclusion:**

DECT parameters IC, normalized IC, *λ*_HU_, and DEI reflect pathologic changes in lymph nodes to a certain extent, and aid for detection of metastatic cervical lymph nodes from OSCC.

**Key Points:**

*• Electron density, iodine concentration, normalized iodine concentration, λ*_*HU*_ *, and dual-energy index values showed significant differences between metastatic and non-metastatic nodes.*

*• Strong correlations were found between iodine concentration, normalized iodine concentration, slope of the spectral Hounsfield unit curve, dual-energy index, and microvascular density.*

*• DECT qualitative parameters reflect the pathologic changes in lymph nodes to a certain extent, and aid for the detection of metastatic cervical lymph nodes from oral squamous cell carcinoma. *

## Introduction

Oral squamous cell carcinoma (OSCC) is the most common pathological type of oral cancer and has a poor prognosis and high mortality rate [1, 2]. Detecting metastasis to cervical lymph nodes directly affects the selection of treatment options and metastasis is the major prognostic factor after treatment [3, 4]. For most nodes without typical internal necrosis or extranodal extension, it is difficult to define metastasis using traditional computed tomography (CT) and magnetic resonance imaging (MRI) [5]. Ultrasonography, which shows good performance in patients with thyroid carcinoma [6], is not a better imaging modality in orofacial malignancies compared with CT [7]. Many new technologies, including magnetic resonance diffusion, magnetic resonance and CT perfusion imaging, and positron emission tomography (PET), have been studied for this purpose [8–10]. However, these imaging techniques cannot provide high specificity and sensitivity simultaneously. Moreover, evaluating the risk of occult neck lymph node metastasis in early-stage patients is still controversial.

Dual-energy CT (DECT) not only provides images similar to single-energy CT but can also be used to perform material decomposition [11]. DECT can calculate quantitative indexes including iodine concentration (IC) and slope of the spectral Hounsfield unit curve (*λ*_HU_). IC analysis in malignant tumors showed positive correlation with microvascular density [12, 13], which can be used for prediction and early diagnosis of lymph node metastasis [14, 15]. DECT can also determine the electron density (ED), effective atomic number (*Z*_eff_), and dual-energy index (DEI) of substances, thus providing more analytic ability for the diagnosis of lymph node metastasis [16, 17]. Recently, DECT-derived IC and *λ*_HU_ have been used to help diagnose metastatic cervical lymph nodes in patients with papillary thyroid carcinoma [18, 19]. IC has also been used to differentiate metastatic head and neck squamous cell carcinoma cervical lymph nodes from normal and inflammatory lymph nodes [20, 21]. However, the utility of ED, *Z*_eff_, and DEI has not been assessed in these studies. To date, few studies have focused on the diagnostic value of DECT parameters in detecting metastasis to the cervical lymph nodes in patients with OSCC.

We hypothesized that metastatic OSCC cervical lymph nodes can be noninvasively characterized using DECT and further validated with radiological, quantitative, and analytical aspects. The purpose of this study was to retrospectively assess the utility of ED, *Z*_eff_, IC, *λ*_HU_, and DEI from a third-generation dual-source DECT in diagnosing metastatic cervical lymph nodes in patients with OSCC.

## Materials and methods

Our study was approved by the Medical Ethical Committee of the Second Xiangya Hospital of Central South University. Given the retrospective nature of this study, the requirement for informed consent was waived. This study was conducted in accordance with the 1964 Declaration of Helsinki and its later amendments.

### Patients

At the Second Xiangya Hospital of Central South University, 267 patients with OSCC were retrospectively identified from November 2019 to June 2021. The inclusion criteria were having undergone radical resection of OSCC and cervical lymph node dissection with available non-contrast and parenchymal phase DECT imaging obtained within 2 weeks before the procedure. The exclusion criteria were images with severe dental streak artifacts, history of other head and neck malignancies, or acute non-specific inflammation.

### Imaging technique

All DECT images were obtained using a third-generation 192-slice dual-source CT scanner (SOMATOM Force; Siemens Medical Solutions). The scanning range was from the skull base to the root of the neck. In order to keep mouth open and therefore reduce dental streak artifacts, patients were asked to bite a bandage during scanning. The scans were performed in non-contrast phase and venous phase while the patients were breathing quietly. The scanning parameters were as follows: A tube voltage, 80 kV; B tube voltage, sn150 kV; CARE kV and CARE Dose 4D modes enabled; collimator size, 192 × 0.6 mm; rotation time, 0.5 s; pitch, 0.8; and matrix, 512 × 512. A double-barrel high-pressure syringe was used to inject the contrast agent iopromide (Ultravist, Shering) through the elbow vein at a flow rate of 3.0 mL/s, with a total amount of 1.2 mL/kg, followed by 30 mL of normal saline. Bolus tracking was used to trigger the scans. The trigger point was placed in the carotid artery at the hyoid level. The trigger threshold of parenchymal phase was 100 Hounsfield unit (HU) and was delayed for 40 s.

### Image analysis

**Fig. 1:**
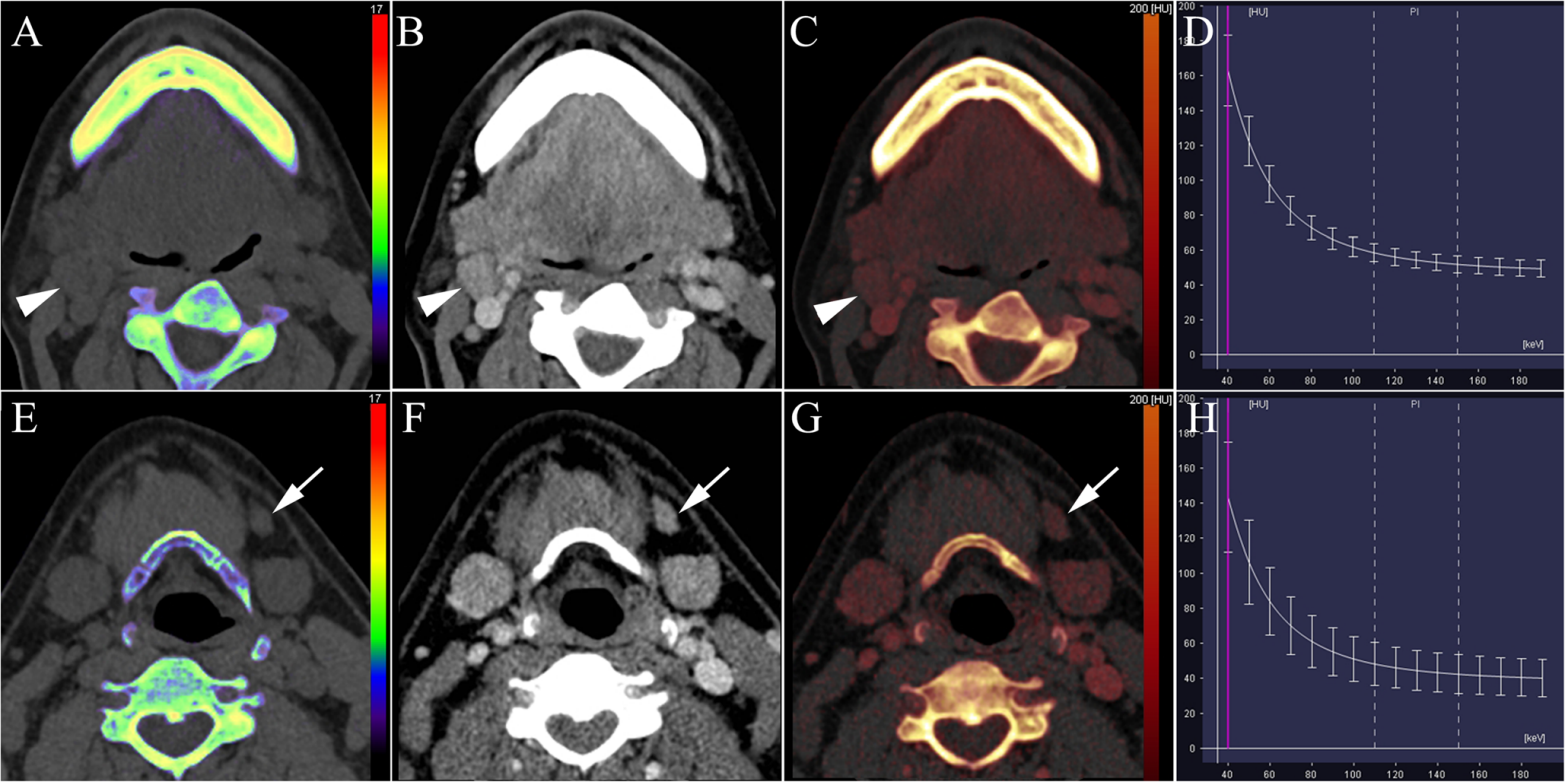
Reconstructed dual-energy images and measurement of quantitative parameters. Unenhanced electron density/*Z*_eff_ map (**A**), linear blending image at HU_40keV_ (**B**), iodine concentration map at parenchymal phase (**C**), and energy spectrum curve (**D**) of a visually metastatic lymph node (arrowhead). Dual-energy parameters and pathologic analysis indicated non-metastasis. Unenhanced electron density/*Z*_eff_ map (**E**), linear blending image at HU_40keV_ (**F**), iodine concentration map at parenchymal phase (**G**), and energy spectrum curve (**H**) of a visually non-metastatic lymph node (arrow). Dual-energy parameters and pathologic analysis indicated metastasis

All the images were automatically transmitted to the post-processing workstation (Syngo Via VB20B, Siemens Healthcare), and non-contrast and parenchymal phase images were reconstructed and analyzed (Fig. 1). The DECT imaging characteristics, including length, width, aspect ratio, necrosis, capsule infiltration, extranodal extension, and aggregation of each lymph node, were recorded. The aspect ratio was calculated as follows:

$$ Aspect\ ratio=\frac{Length}{Width} $$

ED and *Z*_eff_ in non-contrast-enhanced phase, as well as DEI in parenchymal phase, was measured using “Rho/Z” model. Regions of interest (ROIs) on the automatically generated pseudo-color map were manually selected, and values of ED, *Z*_eff_, and DEI were displayed automatically. “Liver VNC” and “monoenergetic” modes were selected respectively to obtain the parenchymal phase IC map and energy spectrum curve at 40~190 keV energy level. The circular or elliptical ROIs were defined at the maximum level of the solid area of the lymph node to measure the IC and *λ*_HU_. The normalized IC was calculated as follows:

$$ Normalized\  IC=\frac{IC_{lymph}}{IC_{vein}} $$

The IC_lymph_ is the IC of the selected lymph node, and the IC_vein_ is the IC of the internal jugular vein at the same level as the lymph node.

The *λ*_HU_ was calculated as follows:

$$ {\lambda}_{HU}=\frac{HU_{40 keV}-{HU}_{100 keV}}{60} $$

where HU_40keV_ and HU_100keV_ are the HU values of the 40-keV and 100-keV monochromatic images, respectively [21].

Measurements were repeated three times for each lymph node, and the mean values were calculated. The cervical lymph nodes in the DECT were marked by a radiologist (Y.-H.L.) with 15 years of experience who was blinded to the pathological results. The level and sequence of each lymph node were recorded according to the 8th edition of the American Joint Committee on Cancer (AJCC) TNM staging [22].

### Surgery

All patients with OSCC underwent complete resection of the primary lesion and neck node dissection of levels I–III ipsilaterally. For patients with suspected or biopsy-proven metastatic cervical lymphadenopathy (levels I–III), extended lymph node dissection (levels IV–VII) was performed as appropriate. Cervical lymph nodes were marked during surgery, with reference to the adjacent structures (tongue, parotid, internal jugular vein, sternocleidomastoid muscle, and others). Lymph nodes that did not achieve one-to-one correspondence between the preoperative DECT and surgery were excluded from this study.

### Pathological analysis

All resected specimens were fixed with 10% formalin solution, embedded in paraffin, and cut into slices. The slices were stained with hematoxylin and eosin and labeled with monoclonal CD34 antibody (GB121693, Servicebio) to detect vascular endothelial cells. Each immunopositive structure (round, oval, and irregular) separated from the other tissue elements was considered a single vessel. Scoring of the microvascular density was performed according to the Weidner counting method [23]. The mean value of microvascular number in the three fields with the highest vascularity (hotspots) was taken as the microvascular density. The greatest vascularization was selected using 200× magnification, and the number of microvessels was counted under 400× magnification. Specimens were assessed by two independent examiners (K.Z. and X.W.) who were blinded to the study using an Olympus microscope (DP72, Olympus Corporation), and the results were averaged.

### Statistical analysis

Statistical analysis was performed using SPSS software (version 22.0; IBM Corp.). Clinically significant variables were identified by comparing the demographic data of patients with or without metastasis, and DECT variables were identified by comparing the ED, *Z*_eff_, IC, normalized IC, *λ*_HU_, and DEI of lymph nodes between patients with and without metastasis. Student’s *t*-test was used to compare the data. The association between DECT parameters and histological characteristics was assessed using Pearson’s rank correlation. Based on the gold standard of pathology, the accuracy, sensitivity, and Youden index of DECT parameters were calculated. The 95% confidence interval (CI) was calculated using the adjusted Wald method. Statistical significance was defined as *p* < 0.05.

## Results

### Study population

**Fig. 2:**
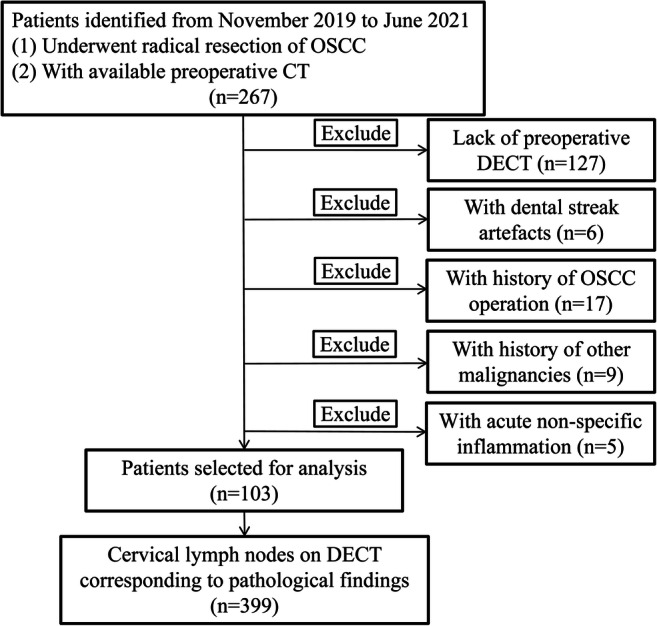
Flow diagram of patient and cervical lymph node inclusion and exclusion criteria

The inclusion criteria were met by 399 lymph nodes from 103 patients with OSCC (Fig. 2). There were 97 men and 6 women, with ages ranging from 31 to 79 years (median, 52 years). Of the 399 lymph nodes, 158 were proven to be metastatic, with an overall metastatic ratio of 39.6% (158/399). In terms of the location of OSCC, there were 50 squamous cell carcinomas of the tongue, 33 in the buccal mucosa, 11 on the floor of mouth, and 9 in the gingiva. The primary site of OSCC did not differ in patients and lymph nodes between metastasis and non-metastasis (both *p* > 0.05). Patients with higher T staging of OSCC were more likely to experience cervical lymph node metastasis than patients with lower T staging of OSCC (*p* < 0.05). The detailed clinical characteristics of the entire cohort are presented in Table 1.

**Table 1:** Comparison of the clinical characteristics of patients and DECT characteristics of lymph nodes between metastasis and non-metastasis

Patients			
Clinical characteristics	Metastasis (*n* = 33)	Non-metastasis (*n* = 70)	*p* values
Age median, range (years)	55.5 (37–70)	52.3 (31–79)	0.102
HPV positive	0 (0%)	1 (1.4%)	0.168
Sex			0.336
Male	30 (90.9%)	67 (95.7%)	
Female	3 (9.1%)	3 (4.3%)	
T category			< 0.001**
T1	0 (0%)	19 (21.1%)	
T2	17 (51.5%)	40 (57.1%)	
T3	14 (42.4%)	11 (15.8%)	
T4	2 (6.1%)	0 (0%)	
Location of OSCC			0.536
Tongue	16 (48.5%)	34 (48.6%)	
Buccal mucosa	10 (30.3%)	23 (32.9%)	
Floor of mouth	7 (21.2%)	2 (2.9%)	
Gingiva	0 (%)	11 (15.6%)	
Lymph nodes			
DECT characteristics	Metastasis (*n* = 158)	Non-metastasis (*n* = 241)	*p* values
T category			< 0.001**
T1	0 (0%)	35 (14.5%)	
T2	52 (32.9%)	111 (46.1%)	
T3	93 (58.9%)	89 (36.9%)	
T4	13 (8.2%)	6 (2.5%)	
Location of OSCC			0.752
Tongue	72 (45.6%)	108 (44.8%)	
Buccal mucosa	24 (15.1%)	82 (34.0%)	
Floor of mouth	62 (39.3%)	17 (7.1%)	
Gum	0 (0%)	34 (14.1%)	
Length (mm)	14.5 ± 6.4	10.0 ± 3.0	< 0.001**
Width (mm)	11.1 ± 5.1	6.8 ± 2.1	< 0.001**
Aspect ratio	1.34 ± 0.24	1.52 ± 0.43	< 0.001**
Necrosis			< 0.001**
Yes	67 (42.4%)	13 (5.4%)	
No	91 (57.6%)	228 (94.6%)	
Capsule infiltration			< 0.001**
Yes	48 (30.4%)	3 (1.2%)	
No	110 (69.6%)	238 (98.8%)	
Extranodal extension			< 0.001**
Yes	24 (15.2%)	1 (0.4%)	
No	134 (84.8%)	240 (99.6%)	
Aggregation			< 0.001**
Yes	33 (20.9%)	0 (0%)	
No	125 (79.1%)	241 (100%)	

*OSSC* oral squamous cell carcinoma, *DECT* dual-energy computed tomography, *HPV* human papillomavirus. **p < 0.01

### Histopathologic analysis

**Fig. 3:**
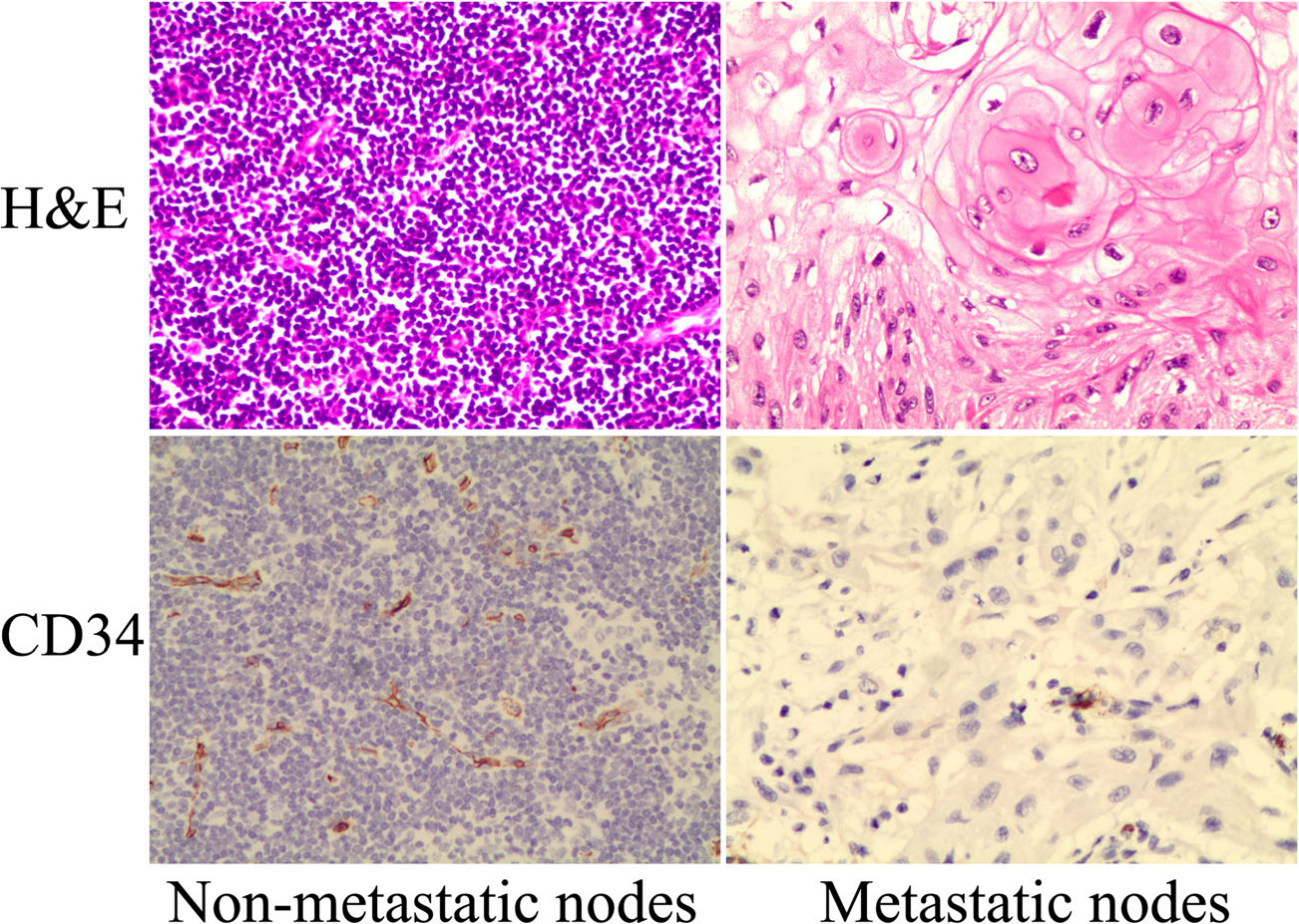
Images of pathological analysis. H&E and CD34 labels of metastatic and non-metastatic cervical lymph nodes (light microscopy, 200×)

Histological analysis showed punctate and flaky necrosis and keratinization developed in the metastatic cervical lymph nodes (Fig. 3). Necrosis was not observed in the non-metastatic group. Analysis of CD34 demonstrated significantly lower mean microvascular density values in the metastatic nodes (27.46 ± 4.48) than in the non-metastatic nodes (46.37 ± 5.21) (*p* < 0.01).

### DECT quantitative analysis

The length, width, and aspect ratio of lymph nodes showed significant differences between metastasis and non-metastasis (all *p* < 0.05; Table 1). The mean ( ± SD) DECT parameters of the metastatic and non-metastatic lymph nodes are summarized in Table 2. There were also significant differences in ED, *Z*_eff_, IC, normalized IC, *λ*_HU_, and DEI between metastatic and non-metastatic lymph nodes (all *p* < 0.01).

**Table 2:** DECT multi-parameter single-variable logistic regression analysis between metastatic and non-metastatic cervical lymph nodes

DECT parameters	Metastatic lymph nodes (*n* = 158)	Non-metastatic lymph nodes (*n* = 241)	*p* values
ED (HU)	36.86 ± 8.32	39.92 ± 6.69	< 0.001**
*Z*_eff_	7.73 ± 0.33	7.76 ± 0.29	0.333
IC (mg/mL)	0.98 ± 0.42	1.55 ± 0.39	< 0.001**
Normalized IC	0.31 ± 0.11	0.51 ± 0.15	< 0.001**
*λ*_HU_	1.30 ± 0.37	1.80 ± 0.46	< 0.001**
DEI	0.011 ± 0.004	0.018 ± 0.005	< 0.001**

*DECT* dual-energy computed tomography, *ED* electron density, *HU* Hounsfield unit, *Z*_*eff*_ effective atomic number, *IC* iodine concentration, *λ*_*HU*_ slope of the spectral Hounsfield unit curve, *DEI* dual-energy index. **p < 0.01

### Correlation analysis

**Fig. 4:**
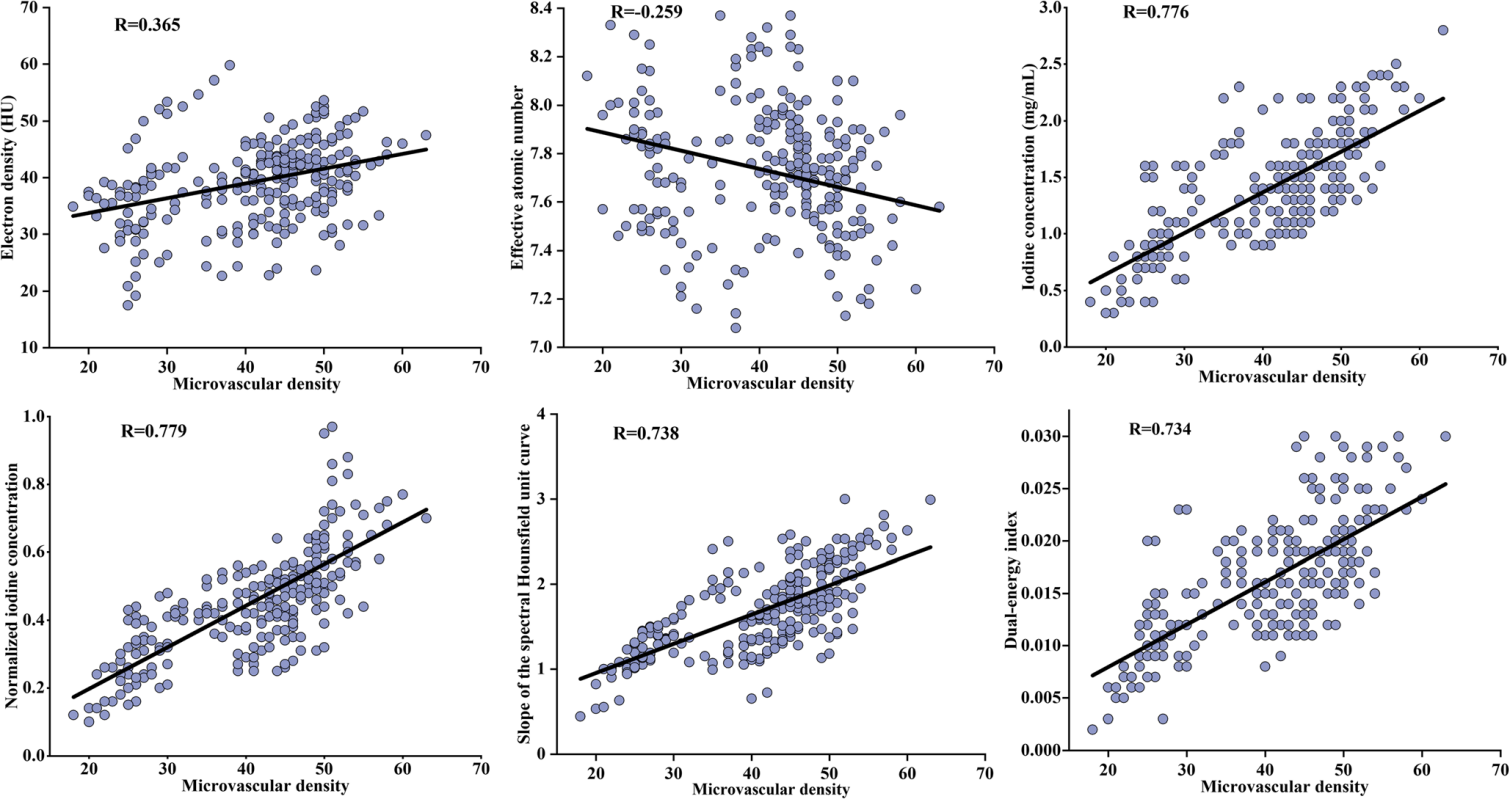
Pearson correlation between DECT parameters and microvascular density

Among DECT parameters, normalized IC has the highest correlation coefficient between microvascular densities (*r* = 0.779), which was slightly better than that between IC and microvascular density (*r* = 0.776; Fig. 4). The *λ*_HU_ and DEI values also showed strong positive correlations with microvascular density (*r* = 0.738 and *r* = 0.734, respectively). However, the ED and *Z*_eff_ values showed weak correlations with microvascular density (*r* = 0.365 and *r* = −0.259, respectively).

### Receiver operating characteristic (ROC) analysis

**Fig. 5:**
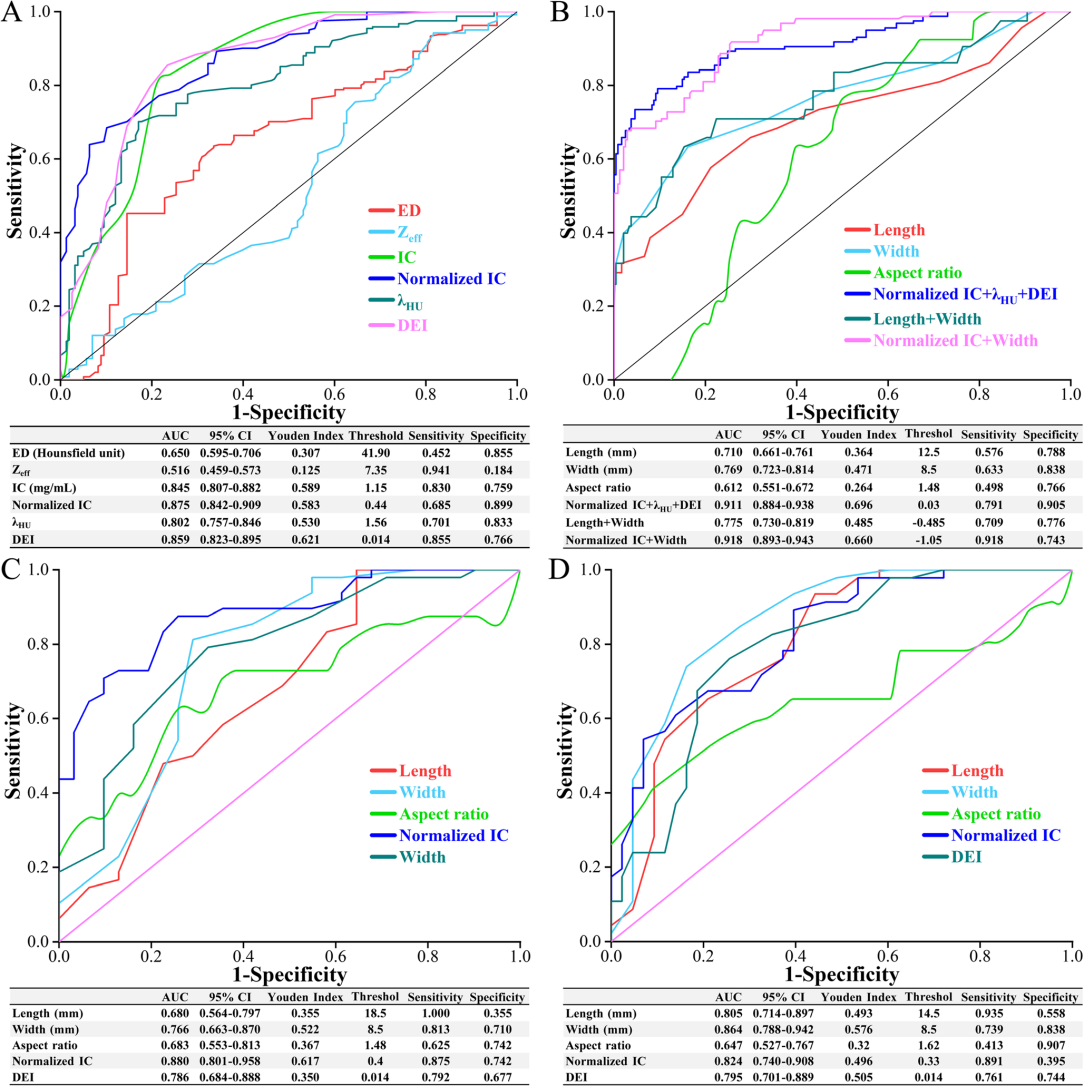
Receiver operating characteristic curve (ROC) analysis. ROC curve and performance of dual-energy CT quantitative parameters (**A**), and characteristics and combination of parameters (**B**) in diagnosing metastatic cervical lymph nodes. ROC curve and performance of parameters in diagnosing metastatic cervical lymph nodes at level IIA (**C**) and level IIB (**D**). ED, electron density; Z_eff_, effective atomic number; IC, iodine concentration; λ_HU_, slope of the spectral Hounsfield unit curve; DEI, dual-energy index; AUC, area under precision-recall curve; CI, confidence interval

The ROC curves and diagnostic ability of ED, *Z*_eff_, IC, normalized IC, *λ*_HU_, and DEI are shown simultaneously on one graph (Fig. 5). The largest area under the curve (AUC) was found in the normalized IC (0.875, 95% CI 0.842–0.909; Fig. 5A), exceeding the AUC for IC and other DECT parameters. The optimal normalized IC threshold value (below 0.44) for diagnosing metastatic nodes had 68.5% sensitivity and 89.9% specificity. The ED, IC, *λ*_HU_, and DEI had the best decision thresholds of 41.90 HU, 1.15 mg/mL, 1.56, and 0.014 respectively. At these thresholds, the sensitivities were 45.2%, 83.0%, 70.1%, and 85.5%, and the specificities were 85.5%, 75.9%, 83.3%, and 76.6%, respectively. However, the AUC for *Z*_eff_ was only 0.516 (95% CI, 0.459–0.573), with a sensitivity of 0.941 and a specificity of 0.184.

In comparison, length, width, and aspect ratio of lymph nodes achieved AUC of 0.710 (95% CI, 0.661–0.761), 0.769 (95% CI, 0.723–0.814), and 0.612 (95% CI, 0.551-0.672), respectively (Fig. 5B).

Combinations of DECT quantitative characteristics were also verified for diagnostic efficiency. The combination of normalized IC and width was more effective than using any one parameter alone and other combinations for the prediction of metastatic lymph nodes, with an AUC, sensitivity, and specificity of 0.918, 91.8%, and 74.3%, respectively (Fig. 5B).

Subanalysis of normalized IC, DEI, and size of nodes in diagnosing metastatic cervical lymph nodes at level IIA (*n* = 89) and level IIB (*n* = 79) was also performed. The largest AUC in diagnosing lymph nodes metastasis at level IIA was found in the normalized IC (0.880, 95% CI 0.801–0.958; Fig. 5C). However, width of lymph nodes had larger AUC (0.864, 95% CI 0.788–0.942; Fig. 5D) than using other parameters for diagnosing metastatic lymph nodes at level IIB.

## Discussion

In this study, the DECT-derived quantitative parameters ED, *Z*_eff_, IC, normalized IC, *λ*_HU_, and DEI were validated to detect cervical lymph node metastasis in patients with OSCC. The ED, IC, normalized IC, *λ*_HU_, and DEI values were significantly different between the metastatic and non-metastatic lymph nodes. The normalized IC achieved best performance in diagnosing metastatic cervical lymph nodes, while combination of normalized IC and width was more effective than using any one parameter alone.

In recent years, few studies on ED and *Z*_eff_ have been reported. The ED is commonly used to calculate the dose in radiation therapy planning with conventional CT. New computerized axial tomography system observed that elements with atomic number (*Z*) can be easily recognized and distinguished one each other. Recently, specific attention is focused on the material decomposition algorithms (two- and three-basis-material decomposition algorithms) and on effective Rho-*Z* methods, which lead to a new clinical practice. The ED varies with the location of the electrons, elemental composition, and structure of the tissue. In this study, metastatic cervical lymph nodes tended to have a lower ED value than normal nodes, with a threshold of 41.85. Our finding is similar to that of Nagano’s study of patients with non-small cell lung cancer [24]. They attributed the lower ED in metastatic mediastinal nodes to the potentially increased connective tissue content. However, a recently published study on colorectal cancers found that ED did not significantly differ between metastatic and non-metastatic lymph nodes [25]. One possible reasons might be differences in the primary tumor, DECT scanner, and nodal size. In contrast to previous studies, ED in non-contrast phase is adopted in our study; it differs between metastatic and non-metastatic lymph nodes, maybe due to the necrosis and keratinization developed in the metastatic cervical lymph nodes.

Substances with different *Z*_eff_ values can be distinguished after material decomposition in DECT [26]. The results of a recent study indicated that *Z*_eff_ could provide not only density but also elemental information of samples [27]. However, in this study, there was no significant difference in *Z*_eff_ between the metastatic and normal nodes. Similar to our findings, Qiu et al found that *Z*_eff_ is not a factor in determining metastatic colorectal cancer lymph nodes [25]. These results differ substantially from those of a 2015 study published by Liu et al, which showed that the *Z*_eff_ value was significantly greater in metastatic lymph nodes than in non-metastatic lymph nodes in patients with papillary thyroid cancer [28]. This may be due to the iodine content in the metastatic lymph nodes in papillary thyroid cancer.

DECT-derived iodine content has an excellent correlation with the true IC and is directly proportional to the amount of iodinated contrast agents in both the intra- and extravascular spaces [29]. Since enhancement in parenchymal phase is due to leakage of contrast agent into the extravascular cavity, the IC in parenchymal phase is more suitable for identifying metastatic lymph nodes [28]. In this study, metastatic lymph nodes showed a lower IC and lower normalized IC in parenchymal phase than non-metastatic nodes, suggesting a lower blood volume or vascular permeability. Our findings are similar to those of studies of patients with squamous cell carcinoma of the head and neck [20] and oropharynx [21]. These results differ substantially from other recently published literature on DECT of thyroid cancer, which showed elevated IC in metastatic nodes [18, 19, 30]. The elevation of IC within metastatic thyroid cancer nodes can likely be explained by the iodine absorption characteristics and the rich blood supply of thyroid tissue. The decrease in IC in our OSCC metastatic squamous cell carcinoma lymph nodes is mainly due to the reduced vascularity and internal necrosis.

The *λ*_HU_ reflects the attenuation in HU of a substance across the 40–140 keV range and is determined by the physical and chemical nature of the substance. In our study, the *λ*_HU_ of metastatic nodes was lower than that of non-metastatic nodes, which is consistent with the trend of IC and normalized IC. Given that *λ*_HU_ depends greatly on the iodine content within the tissue, this tendency was reasonable. This result is also consistent with those of previous studies of oropharyngeal squamous cell carcinoma [21] and papillary thyroid carcinoma [30, 31].

Moreover, DEI was found to be another predictor of lymph node metastasis. DEI calculation has been used in phantom and forensic case studies for the classification of bullets [32, 33]. Our study revealed a lower DEI value in OSCC metastatic lymph nodes than benign lymph nodes correlating with pathological finds. It is probably due to pathological changes in OSCC-related necrosis and decreased microvascular density. A previous study of patients with rectal cancer showed a contrary result [34], but the reason for the increase in DEI was not discussed in that study. Compared with the previous study, we had a much higher AUC (0.859 vs. 0.697).

This study had several limitations. First, due to the limited number of lymph node samples, it was difficult to avoid selection bias and perform further subgroup analysis. Second, the validation was based on quantitative parameters derived from the region of interest of the lymph nodes. The shape, size, extranodal spread, and location of cervical lymph nodes that would further increase the diagnostic effectiveness [35] were not considered. Future studies that combine these features and DECT-specific parameters are warranted. Third, 2 dual-source acquisitions on the same patient were performed. Although the radiation dose is within the safe range, adoption of normalized IC and width and removing ED and *Z*_eff_ in non-contrast phase would ensure diagnostic value while reducing X-ray exposure. Fourth, other imaging modalities, including PET, MRI, and ultrasonography, were not used in our study for the comparison of diagnostic efficacies.

## Conclusion

In conclusion, the values of normalized IC, *λ*_HU_, and DEI reflect the changes in pathogenesis within lymph nodes to a certain extent. They may help clinicians better identify metastatic cervical lymph nodes in patients with OSCC and aid clinical decision-making.

## Abbreviations

AJCC: American Joint Committee on Cancer
AUC: Area under the curve
CT: Computed tomography
DECT: Dual-energy CT
DEI: Dual-energy index
ED: Electron density
IC: Iodine concentration
MRI: Magnetic resonance imaging
OSCC: Oral squamous cell carcinoma
PET: Positron emission tomography
ROC: Receiver operating characteristic
ROI: Region of interest
*Z*_eff_: Effective atomic number
*λ*_HU_: Slope of the spectral Hounsfield unit curve

## Declarations

### Guarantor

The scientific guarantor is Ya-Jun Li.

### Statistics and biometry

No complex statistical methods were necessary for this paper.

### Methodology

• Retrospective

• Diagnostic study

• Single-center research
